# Design and fabrication of a nerve-stretching device for *in vivo* mechanotransduction of peripheral nerve fibers

**DOI:** 10.1016/j.ohx.2020.e00093

**Published:** 2020-02-07

**Authors:** Muhammad Sana Ullah Sahar, Matthew Barton, Geoffrey Tansley

**Affiliations:** a School of Engineering and Built Environment, Griffith University, Australia; b Clem Jones Centre for Neurobiology and Stem Cell Therapies, Griffith University, Australia; c Menzies Health Institute Queensland, Griffith University, Australia; d School of Nursing and Midwifery, Griffith University, Australia

**Keywords:** Nerve stretch growth, Axonal growth, *In-vivo* nerve stretching, Nerve stretcher, Nerve lengthening

## Abstract

The potential of peripheral nerves to regenerate under the effect of axial tensile forces was not previously extensively explored due to the lack of capabilities of translating *ex vivo* axonal stretch-growth to *in vivo* studies, until the development of a *nerve stretcher*. The *nerve stretcher*, which we have designed and manufactured recently, is a device that uses a controlled amount of axial tensile force (vacuum/negative gauge pressure) applied directly to a sectioned peripheral nerve *in vivo* to expedite nerve regrowth rate. Using this platform, a series of experiments was carried out to observe the effect of *in vivo* axial stretch on axonal lengthening. During these experiments, a few challenges necessitated redesigning the device like a sudden loss of stretching force due to vacuum leakage, erroneous feedback from vacuum sensor due to sensor drift, and inability to control and operate the device remotely. Here we present an improved design of the *nerve stretcher* along with its integration with a state-of-the-art online vacuum monitoring facility to control, collect, process, and visualize negative gauge pressure data in real-time.

## Graphical abstract

The basic working principle of a nerve stretcher along with an illustration of its usage for a nerve-stretch-growth experiment. the black lines represent electrical circuitry, while blue lines represent the vacuum path. The basic working principle of a *nerve stretcher* along with an illustration of its usage for a nerve-stretch-growth experiment. The black lines represent electrical circuitry, while blue lines represent the vacuum path.

**Figure:**
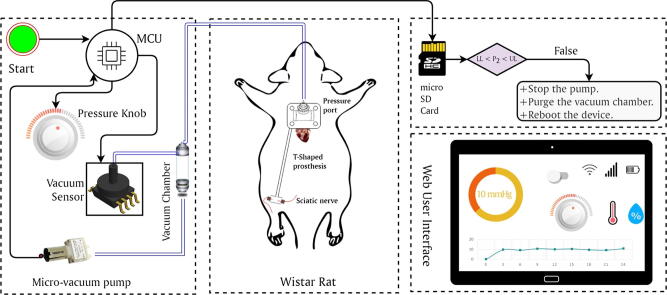

**Specifications table**

**Table:** 

Hardware name	Nerve Stretcher V2
Subject area	• Engineering and Material Science • Neuroscience
Hardware type	• Other: Axonal Stretch Growth
Open Source License	Creative Common – Attribution – ShareAlike 3.0
Cost of Hardware	$240 (AUD)
Source File Repository	https://data.mendeley.com/datasets/2g6xgyvm4d/3

## 1 Hardware in context

Among peripheral nerve injuries, neurotmesis involves the complete severing of nerve fibers, and hence, invariably warrants surgical intervention to bridge the gap between the resected ends; however, even with the aid of modern surgical techniques, the regenerative period may span months to years [1]. The slow rate of nerve growth can often lead to long-term disability and contribute to poor quality of life for the patient. The devastating effects of delayed nerve regeneration can be avoided by increasing the rate of nerve regeneration. *In vitro*, the growth rate of regenerating nerve fibers can be facilitated by stretching them mechanically [2]. This idea of axonal stretch-growth, *ex vivo*, at the tissue level under the effect of purely mechanical tensile forces has been supported by multiple studies [3], [4], [5], [6], [7]. The stretch-growth of nerve fibers, although practically possible *ex vivo*, is more challenging *in vivo*, due to the complexities of applying stretching forces directly to transected nerve ends *in situ*. Recently a method has been proposed for *in vivo* nerve stretch-growth by using a controlled amount of vacuum developed by a *nerve stretcher* and applied directly to nerve stumps [8]. In the current paper, an improved design of the *nerve stretcher* has been presented along with its manufacturing and performance optimization. The device presents itself as a tool to increase the growth rate of a peripheral nerve associated with stretch and is aimed at reducing the inherent limitations of peripheral nerve regeneration after surgery following nerve trauma. Furthermore, it can be used to study mechanotransduction of peripheral nerves *in vivo*.

## 2 Hardware description

The current design brings many components of the device from the previous version of the device, along with the replacement of micro-controller (MCU) with one having built-in WiFi connectivity, an improved vacuum sensor, and a man-machine (graphical user) interface to interact and control the device as well as to monitor stretching force (negative gauge pressure) in real-time. The new platform is also equipped with a monitoring system to study the animal’s behavior remotely during experiments. These improvements brought a set of new features to the *nerve stretcher* and made it possible to run it without having direct physical access to it over an extended period.

**Fig. 1:**
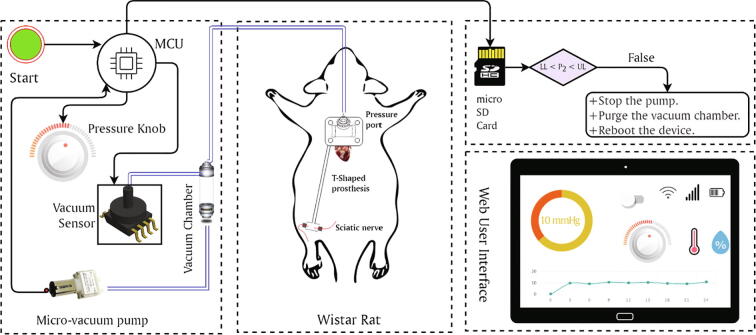
An illustration of the working of a *nerve stretcher*. MCU is responsible for controlling the device and running the vacuum pump based on the feedback of the vacuum sensor. The desired amount of negative gauge pressure can be achieved in the vacuum chamber by turning the pressure knob to the desired value. MCU also keeps a record of the amount of vacuum in the vacuum chamber and is programmed to shut down the device and release vacuum if pressure increases than the desired pressure. The device can be fully controlled using a web-based user interface. Note: The black lines represent electrical circuitry, while blue lines represent the vacuum path. (For interpretation of the references to colour in this figure legend, the reader is referred to the web version of this article.)

Note: All pressures mentioned in this article are negative, relative to standard atmospheric pressure.

The electronic circuit of a *nerve stretcher* consists of a micro-controller (Espressif Systems, CO., LTD CN), vacuum sensor (NXP Semiconductors, Inc. NL), solenoid valve, micro-vacuum pump (Skoocom Electronic Co., Ltd, CN), mechanical relay (TE Connectivity Ltd. CH), and a generic graphics display. The vacuum pump generates negative gauge pressure in a vacuum chamber (KCI Medical Ltd, IRL) which is monitored by the vacuum sensor and is maintained and controlled at any desired level by the micro-controller. The micro-controller is programmed through a custom control module, written in C language, and having the ability to transmit the device’s metrics over a WiFi connection to a remote *web user interface* which allows control of the device and monitoring of its output in real-time. Fig. 1 shows an illustration of the working of a *nerve stretcher*.

## 3 Design files

A description of the design files that were used to program the nerve stretcher is provided in Table 1. All these files can be downloaded from the Mendeley data repository (https://data.mendeley.com/datasets/2g6xgyvm4d/3).

**Table 1:** Design files summary.

Filename	File type	Open source license	Location of the file
Firmware.bin	A source code to run the nerve stretcher.	Creative Commons - Attribution - ShareAlike 3.0	Main Repository
GerberFiles.zip	Gerber files to manufacture printed circuit board of the nerve stretcher.		
Schematic.pdf	A device circuit schematic.		
comPort.ps1	A script to find available COM ports.		
Flasher.bat	A script to upload firmware to MCU.		
getEssentials.ps1	A script to download web-ui.		
PiCamX.sh	A script to set up a camera for monitoring.		
deviceAccessPage.bat	A script to access the homepage of the nerve stretcher.		

## 4 Bill of materials

Table 2 shows the bill of the material used in the production of a nerve stretcher. (Note: The manufacturing cost can significantly be reduced by sourcing components online and/or recycling parts salvaged from previous projects.)

**Table 2:** Bill of materials (CE = Core Electronics AU, E14 = Element 14, Generic = Purchases made from online stores).

Designator	Component	Qty.	Cost per unit (AUD)	Source of materials	Material type
Electronics					
P1, P2, P3, P4	Male header pins	4	$0.3	Generic	Metal
S1	On/Off Pushbutton	1	$0.5	Generic	Electronics
S2	Tactile button switch	1	$0.13	Generic	Electronics
K1, K2	Relay NO type, 3 V, 10A	2	$1.18	E14	Electronics
R1, R2, R3, R4	Resistor 10 KΩ	4	$0.05	E14	Ceramics
R5, R7	Resistor 1 KΩ	2	$0.05	E14	Ceramics
R6, R8	Resistor 120 Ω	2	$0.05	E14	Ceramics
C1, C3	Capacitor 0.33 µF	2	$0.07	E14	Ceramics
C2, C4, C5, C6	Capacitor 0.1 µF	4	$0.07	E14	Ceramics
C7	Capacitor 0.01 µF	1	$0.07	E14	Ceramics
C8	Capacitor 1 µF	1	$0.07	E14	Ceramics
C9	Capacitor 470 pF	1	$0.07	E14	Ceramics
D1, D2, D3	1 N4001 Diode	3	$0.15	Generic	Ceramics
J1	DC power connector	1	$1.37	E14	Electronics
U1	Voltage regulator LP2950cz-3.0	1	$1.57	E14	Electronics
U2	Voltage regulator L78L33acztr	1	$1.98	E14	Electronics
U3	MCP3201 12-bit ADC	1	$4.89	E14	Electronics
U4	MCU ESP8266 ESP-12F	1	$5.48	Generic	Electronics
Q1, Q2	Transistor BC337	2	$0.76	Generic	Ceramics
LED1, LED2	5 mm Red LEDs	2	$0.1	Generic	Glass
Hardware					
Valve	Solenoid valve (NC) 3 V DC	1	$13.45	Generic	Metal
OLED	0.96″ Graphic display SSD1306	1	$6.89	Generic	Ceramics
Pump	Micro vacuum pump SC3101PM	1	$3.05	Ali Express	Metal
Sensor	Vacuum sensor (MP3V5050V)	1	$26.18	E14	Polymer
Shunt	Jumper – 2 Pin, pitch = 2.54 mm	1	$0.48	CE	Plastic
Solder Wire	Solder wire 0.6 mm		$3.45	Generic	Metal
Canister	Vacuum Canister (300 ml)	1	$15	KCI Medical	Plastic
Tubing	Silicone tubing (2 m)	1	$5	Generic	Polymer
SBC	Raspberry Pi 4 (4 GB RAM)	1	$80.89	E14	Electronics
SD card	Micro SD card 16 GB	1	$15	Generic	Plastic
Power adaptor	Power adaptor (5.1 V, 3 A)	2	$15	Generic	Electronics
USB to TTL	USB to Serial Converter	1	$8	Generic	Electronics
Webcam	USB webcam	1	$7.5	Ali Express	Electronics
PCB	PCB Manufacturing	1	$20	JLCPCB	Plastic

## 5 Building instructions

**Fig. 2:**
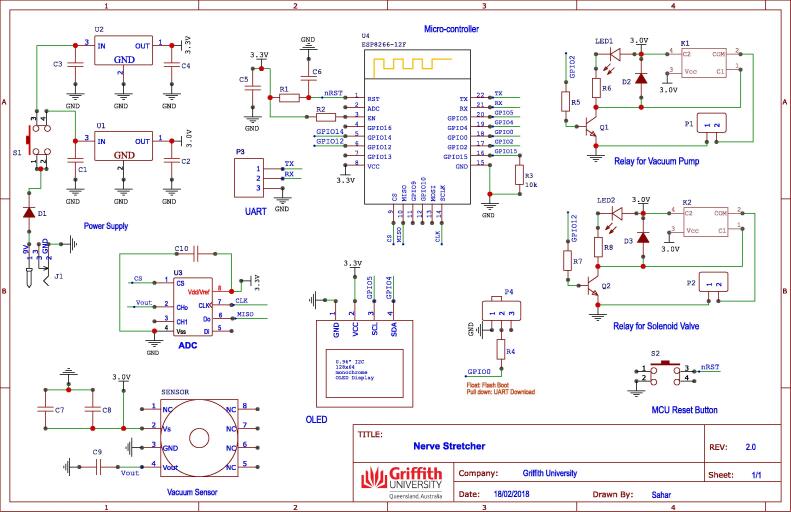
A circuit schematic designed in EasyEDA.

The electronic circuit of the *nerve stretcher* was designed using EasyEDA (https://easyeda.com/editor)- a web-based electronic design software. The free version of this software is sufficient for designing a circuit as it allows easy access to an extensive library of electronic components to choose from. Moreover, it also allows converting design schematic to board schematic, and generating gerber files necessary for printing permanent circuit board (PCB). The symbols and footprints of the required electronics parts listed in Table 2 were picked from EasyEda library and placed on to its canvas, as shown in Fig. 2. The connections between the components were made according to the circuit schematic. The design schematic was converted to board schematic board, and the electrical connections were made using *autorouter* facility of the EasyEda. The board schematic was inspected for DRC (design rule check) errors, and then gerber files, necessary to machine a PCB, were generated. The gerber files were examined for any possible defects using gerber viewer software (http://gerbv.geda-project.org) and then sent for PCB manufacturing (JLPCB, https://jlcpcb.com).

**Fig. 3:**
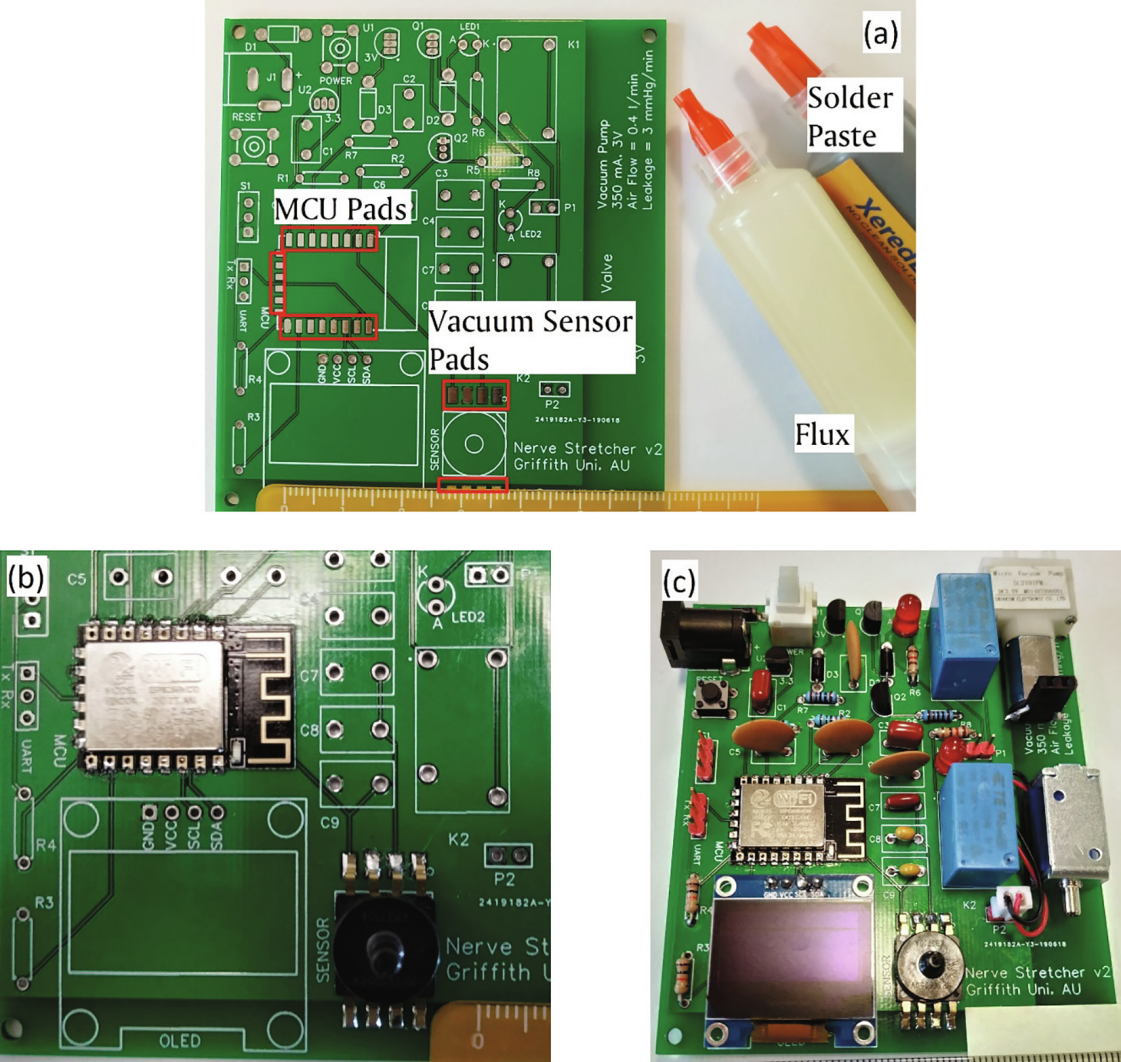
(a) A PCB is showing metallic pads of the MCU and vacuum sensor. (b) SMD components are soldered using SMT soldering technique. (c) A PCB with all of its components soldered on it.

The device building process started with soldering electronic components on the PCB at their designated places. Through-hole soldering technique (THT) was used to solder components except for surface mount devices (SMD), MCU and vacuum sensor, which were soldered using surface mount technique (SMT). Additionally, electrical wires were soldered to the terminals of the micro-vacuum pump and solenoid valve, to make them get connected conveniently to their respective male-header pins (P1 and P2). For SMD soldering, the individual pads of the components on the PCB, shown in Fig. 3(a), were cleaned with isopropyl alcohol (Diggers, AU). Subsequently, a smooth coating of solder-flux was applied on the pads, followed by a smooth layer of solder-paste. The pads were then heated gently with the tip of a soldering iron (Tip temperature: 250 °C) to uniformly deposit the solder paste on them. The flux facilitated the depositing of solder paste on the pads and helped to remove oxidation from the metallic surfaces to be joined. During soldering SMD components, soldering paste was pasted in such a way that it did not exceed the area of the individual pad. PCB tweezers were used to pick and place the components on their corresponding pads, and hot air was blown directly on them using a hot-air gun. The remaining parts were placed on their designated location on the PCB and were soldered using THT soldering. Direct contact with soldering iron tip was avoided. The PCB was then cleaned using a banister brush soaked in isopropyl alcohol, and latex gloves were used for protecting skin against alcohol.

## 6 Software description

The program to run the *nerve stretcher* was written and compiled in an *Arduino integrated development environment* (IDE). The web-based user interface (web-ui) to visualize the device’s metrics and control its attributes was made by using an open-source internet-of-things (IoT) platform (ThingsBoard, Inc., US). All the files and scripts mentioned in these instructions can be downloaded from the Mendeley data repository except where stated.

Note: A laptop/PC running *Microsoft Windows* is required to follow the instructions given below.

### 6.1 Programming the nerve stretcher

To upload the firmware to the MCU of the *nerve stretcher*, the following steps were performed chronologically.

**Fig. 4:**
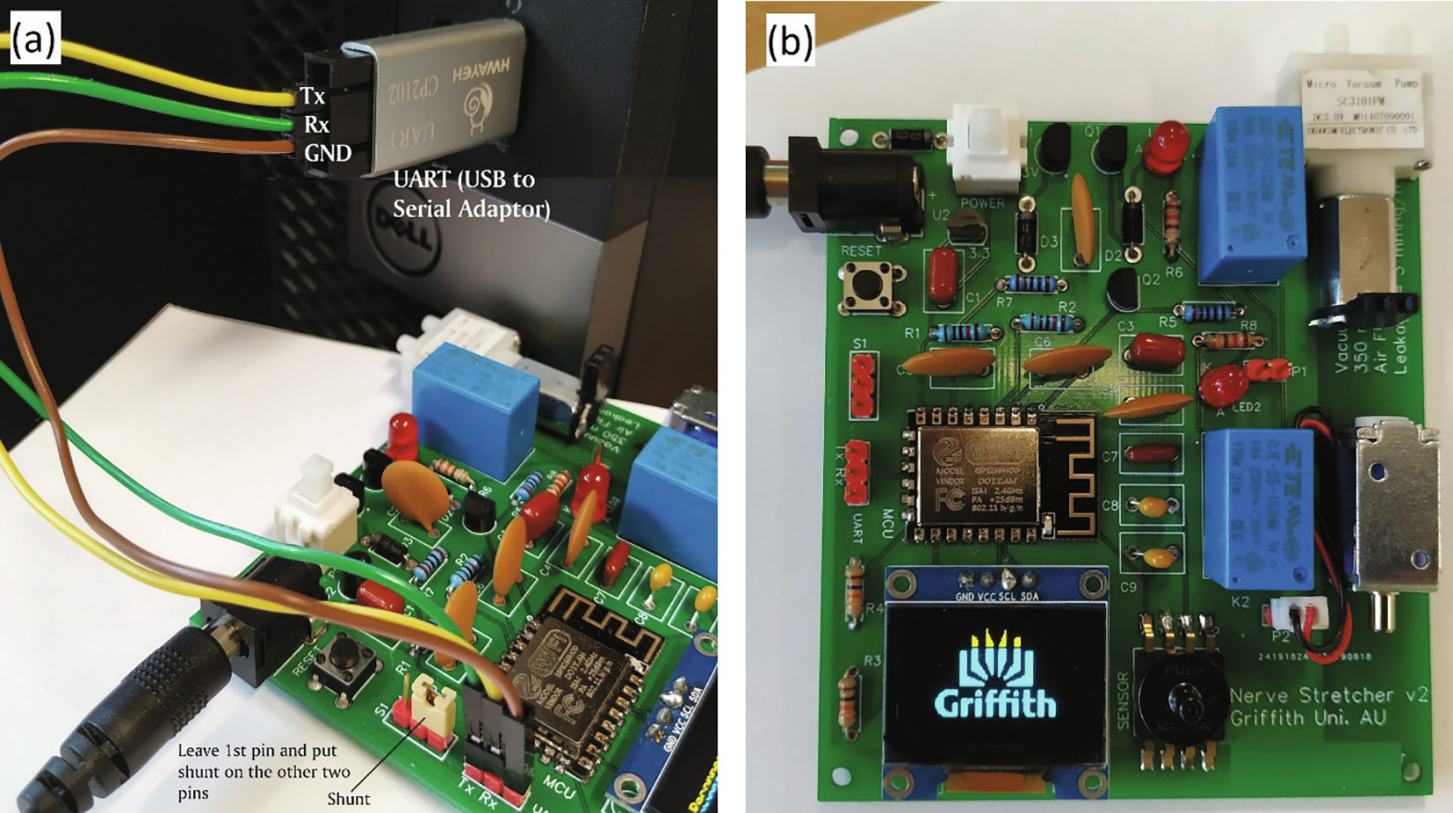
A nerve stretcher is showing (a) configuration required to upload the firmware (b) and a start-up logo during power-on.
• A *serial converter* was connected to the *nerve stretcher* using UART interface at P3. TX, RX, and GND of the serial converter were connected to RX, TX, and GND of the *nerve stretcher*. The serial converter was then inserted into the female USB port of the laptop, as shown in Fig. 4(a).
• The *nerve stretcher* was supplied with power through its power jack, and the power button was pressed.
• A communication port scanner script (*comPort.ps1*) was download and run on the laptop using windows PowerShell which displayed a message box with a *port number* that is required in the next step.
• On the laptop, a script *Flasher.bat* and *nerve stretcher’s* firmware (*Firmware.bin*) were downloaded and the script *Flasher.bat* was run by double-clicking on it to upload firmware to the MCU.
• The serial converter and the 2-pin-shunt were removed, and the *nerve stretcher* was restarted.

• Python 3.7.4 (*https://www.python.org/ftp/python/3.7.4/python-3.7.4.exe*) was installed on a laptop and then *esptool*, a python-based utility to communicate with the bootloader in an MCU, was downloaded and installed by typing “*pip install esptool”* in windows command prompt. The laptop was restarted after completing this step.
• A 2-pin shunt was put on the two pins of P4 terminal, leaving the first pin next to the reset button (S2) untouched as shown in Fig. 4(a).

### 6.2 Programming the Raspberry-Pi

A micro-SD card the with the latest Raspbian operating system (Raspbian Buster Lite) using an image flasher (etcher, https://www.balena.io/etcher). A file (ssh), provided in the repository, was added to boot directory (/boot) of the micro-SD card to allow the incoming connection from laptop to Raspberry-Pi (R-Pi). The R-Pi was powered-on using a universal power adaptor with the micro-SD card inserted into its SD-card slot and an ethernet cable connecting R-Pi to an internet modem. The internet protocol address (IPv4) of the R-Pi was obtained from the administration homepage of the internet modem/router to which it was connected. The command-line interface (CLI) of the R-Pi was accessed on the laptop using PuTTY software (https://www.putty.org). The default username and password (*pi*/*raspberry*) were used to access R-Pi. After logging-in, Wi-Fi connection settings were adjusted by using the following commands;

• sudo wget https://raw.githubusercontent.com/msanaullahsahar/nestv2/master/wifi-settings.sh
• sudo chmod 755 wifi-settings.sh
• sudo ./wifi-settings.sh

### 6.3 Installation of Thingsboard platform

An open-source IoT platform necessary to display the web-based user interface of the *nerve stretcher* was installed on R-Pi. The installation of this software was achieved by downloading a script (*installTB.sh*) from the repository, making it executable, and finally running it by using the commands given below. The installation process was fully automatic, and a web-link was displayed at the end of the installation process to access Thingsboard platform. Finally, a port number 8080 was exposed to the outer web by accessing the router’s homepage (also known as port forwarding) to allow inbound non-local traffic to reach Thingsboard IoT platform.

• sudo wget https://raw.githubusercontent.com/msanaullahsahar/nestv2/master/installTB.sh
• sudo chmod 755 installTB.sh
• sudo./installTB.sh

#### 6.3.1 Importing web-user interface

A modular web-based user interface (web-ui), designed in *JSON* (Javascript object notation) to control the *nerve stretcher*, was imported in Thingboards IoT platform. A script (*getEssentials.ps1*) was downloaded on the laptop and run by right-clicking on it and then choosing “*Run with PowerShell*”. This process generated two files *dashboard.json* and *myDevices.csv* that needed to be imported in later steps. The IoT platform was accessed on the laptop by its URL and default credentials were used for signing on the platform (*Username: tenant@thingsboard.org, Password: tenant*) along with the following steps;

• By selecting devices on the menu bar, two new devices were created by clicking on the plus sign in the bottom of the right-hand side of the screen and importing myDevices.csv file. On import configuration step, both checkboxes were removed, and on select columns type option, column type was chosen as Name, Type and Access token for first, second and third row respectively. Finally, the process was completed by clicking the ok button.
• The web-ui was imported by clicking on dashboards on the menu bar, hitting the plus sign, selecting import dashboard option, choosing the web-ui file (dashboard.json) downloaded earlier, and finally clicking on import button.
• On the newly appeared window (Configure aliases), both aliases were edited one by one. For both cases filter type was chosen as a single entity, type was chosen as a device, and finally, for Nerve Stretcher and Image Counter, devices were chosen as Nerve Stretcher and Image Counter respectively. The configurations were saved by pressing the save button, which completed the web-ui importing process. The web-ui was then accessed by clicking on the newly imported dashboard card (nerve stretcher dashboard).

#### 6.3.2 Online monitoring system

The aim of online monitoring of animals during their post-operative period was to study their behavioral responses to stretch on their sectioned nerves. The online monitoring system only requires connecting the video camera to the R-Pi along with an installation of a script provided in the repository. The video camera, having a standard male USB-type-A port, was interfaced directly with R-Pi using one of its four standard female USB-type-A port. The *Motion software* (*https://motion-project.github.io/index.html*) was then installed to control the webcam, and images were streamed to the web-ui using *iframe* attribute of hypertext markup language. Unfortunately, even taking a few images/frames at regular intervals resulted in a collection of a large number of images that caused the R-Pi to run out of space on its SD card. This challenge was addressed by deleting the images from the image directory at regular intervals using a time-based job scheduler. The information about the number of images in the image directory was sent to the web-ui by message queuing telemetry transport. A script, *PiCamX.sh*, was downloaded, and the following commands were used to install the online monitoring system.

• sudo chmod a + x PiCamX.sh
• sudo ./PiCamX.sh

### 6.4 Final tuning

The *nerve stretcher* was powered-on, and a script (*deviceAccessPage.bat*) was downloaded on the laptop. The laptop was then allowed to connect to a WiFi network- *nESt-V2*, an access point created by the *nerve stretcher*, and the *nerve stretcher’s access point connection page* was accessed by double-clicking the *deviceAccessPage.bat*. On this page, WiFi configurations for the *nerve stretcher* were performed by selecting a *WiFi network* to which the *nerve stretcher* was intended to connect to, supplying password of the WiFi/router/modem and then replacing *RaspberryPi-IP-Address* with the real IP address of R-Pi. After saving the WiFi credentials, the *nerve stretcher* was restarted again, and it displayed a start-up logo, as shown in Fig. 4 (b), indicating that it was ready to use.

## 7 Operation instructions

### 7.1 Device setup

**Fig. 5:**
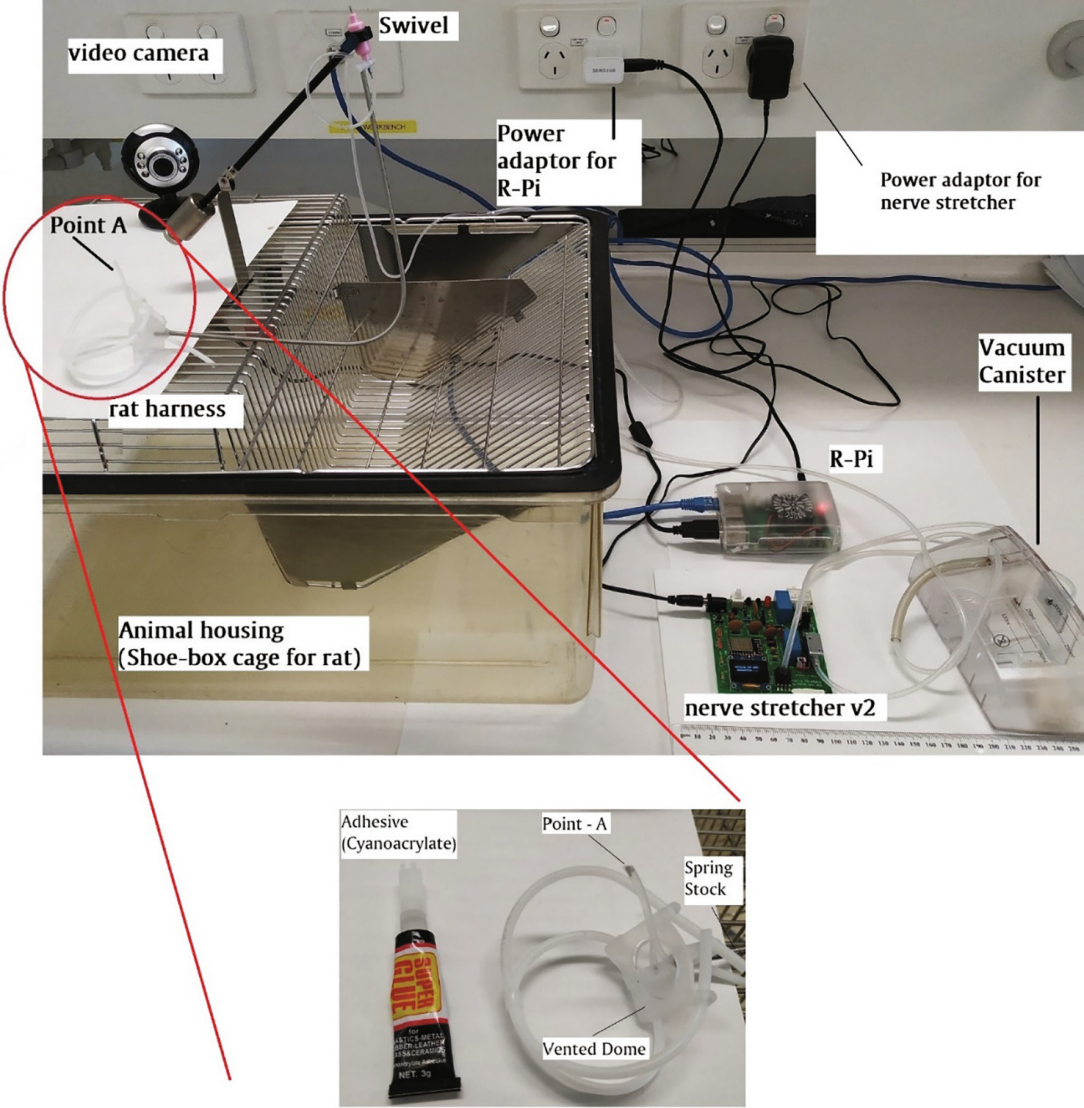
An experimental setup for testing the performance of the *nerve stretcher*.

The *nerve stretcher* was scrutinized before maiden testing for any possible electrical misconnection, and to ensure that if the vacuum chamber was connected correctly to the suction port of the micro-vacuum pump. The silicone tubing connecting the vacuum pump to the vacuum sensor port was made restriction free. The silicone tubing connected to the swivel and passing through spring was closed at its endpoint A, as shown in Fig. 5. The *nerve stretcher* was supplied with power using its respective power adapter which must not be rated higher than 5 V, 2A as it had no overvoltage protection. As a precautionary measure, it was ensured that the shunt connector was not set to flash mode.

### 7.2 Device operation

**Fig. 6:**
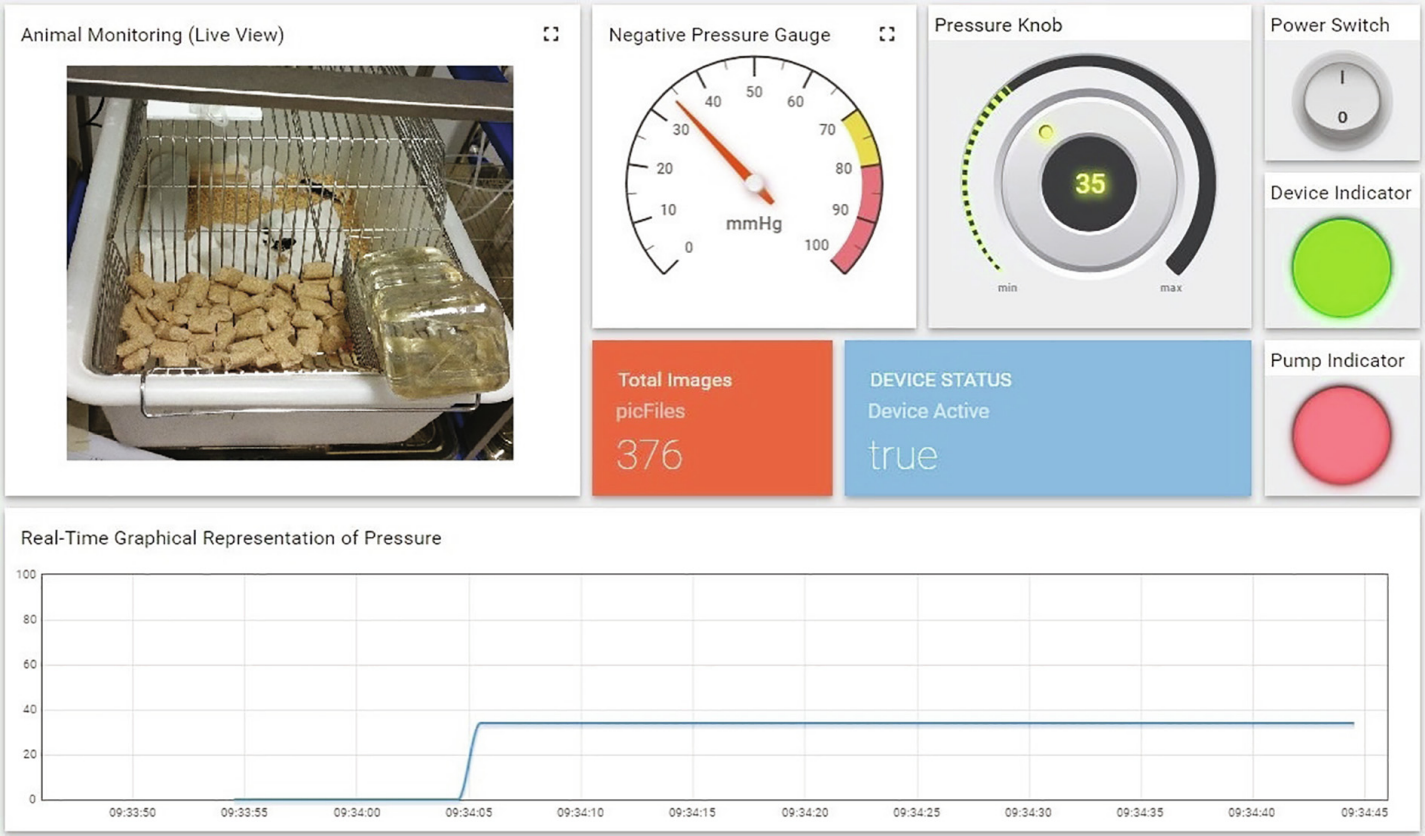
A web-ui of the *nerve stretcher* showing a real-time view of stretching force (vacuum) and device controls.

On pressing the power switch down on the *nerve stretcher*, the solenoid valve having normally-closed-type architecture opened automatically for five seconds, allowing the air to enter in the vacuum chamber. The device’s web-ui was accessed on the laptop. Meanwhile, the *nerve stretcher’s* screen started displaying 0.0 mmHg as a reference pressure, followed by a startup logo which was an indication that the device had been set up correctly. A desired amount of vacuum in the vacuum chamber was achieved by changing the value of reference pressure using the pressure knob on the web-ui. The micro-vacuum pump then started creating a vacuum inside the vacuum chamber, and its quantity was measured and sent continuously by the vacuum sensor to the MCU. The MCU beside running the micro-vacuum pump based on the vacuum sensor’s feedback was actively transmitting the device’s metrics to the web-ui, as shown in Fig. 6.

### 7.3 Calibration, maintenance, and troubleshooting

The *nerve stretcher* being a state-of-the-art technology was equipped with a factory calibrated and temperature compensated vacuum sensor for providing a positive electrical output up to 50 kPa. As the solenoid valve was programmed to allow the air to enter the vacuum chamber to set a reference pressure of 0 mmHg for the vacuum sensor at the device’s startup, it greatly eliminated manual calibration of the vacuum sensor. The MCU was also allowed to restart/turn-off the *nerve stretcher* if the vacuum exceeded the desired pressure (±10 mmHg) for 5 min or if there was a continuous leakage in the system.

## 8 Device performance validation

**Fig. 7:**
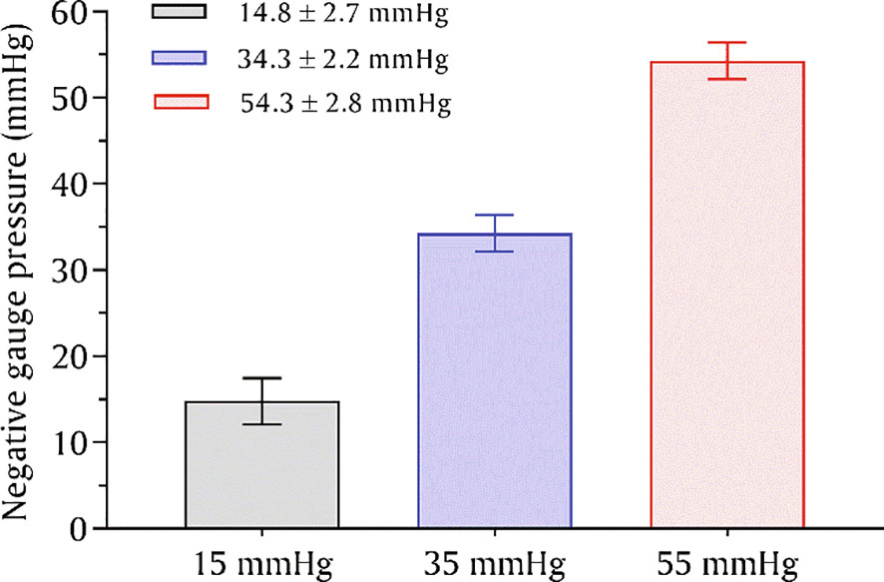
A graph showing the performance of the *nerve stretcher* without leakage at 15, 35, and 55 mmHg, respectively.

The performance of the device was tested by mirroring the previous experimental setup [8] but without using any rat. The vacuum chamber was connected to the micro-vacuum pump, solenoid valve, and vacuum sensor through its input ports, while the output port of the vacuum chamber connected to the top-port of swivel via a silicone tubing. A second silicone tubing connected to the lower-port of swivel, passing through a spring stock, and finally exiting in a vented dome reaches to the point of application (Point *A* in Fig. 5). The spring protects the silicone tubing from animal chewing and transmits torque to the swivel which was mounted on a single-axis counter-balanced lever (Instech Lab Inc., USA). The end of the silicone tubing at *point-A* was fully closed using cyanoacrylate. The nerve stretcher was connected to the wall power supply via its power adaptor, and the web-ui of the nerve stretcher was accessed on a laptop. On the web-ui, once the *device status* changed from *false* to *true,* the *power switch* was toggled to the *ON* position. After that, the performance of the *nerve stretcher* was tested at three representative levels of vacuum, namely 15 mmHg, 35 mmHg, and 55 mmHg. The pressure was set using the pressure knob, as shown in Fig. 6. The device was operated continuously at each level for five minutes. The sensor data (vacuum/negative gauge pressure) was retrieved from the database of the IoT platform and presented in graphical form, as shown in Fig. 7. The mean pressure was found to be 14.8 ± 2.7 mmHg, 34.3 ± 2.2 mmHg, and 54.3 ± 2.8 mmHg, respectively.

**Fig. 8:**
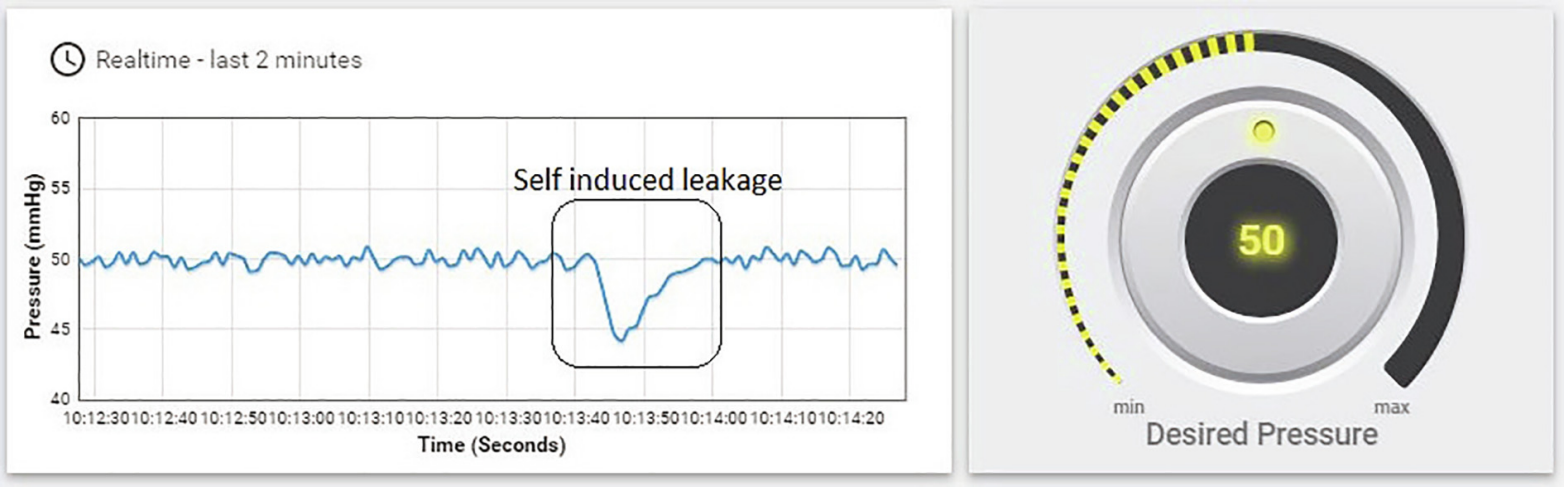
A graph is showing the ability of the *nerve stretcher* to detect leakage of the system.

A further assessment was conducted to ensure the *nerve stretcher’s* capability to detect and report leakage. The *nerve stretcher* was run to maintain a pressure of 50 mmHg, and once the pressure stabilized, leakage was induced by puncturing the silicone tube at *point A* with a sharp needle and immediately closing it with a metallic clamp. The leakage was detected by the system and reflected in the form of a sharp trough, as shown in Fig. 8. As the performance of the *nerve stretcher* can be observed in real-time, it made it easier to detect when pressure was lost, necessitating attention.

### 8.1 System leakage rate

**Fig. 9:**
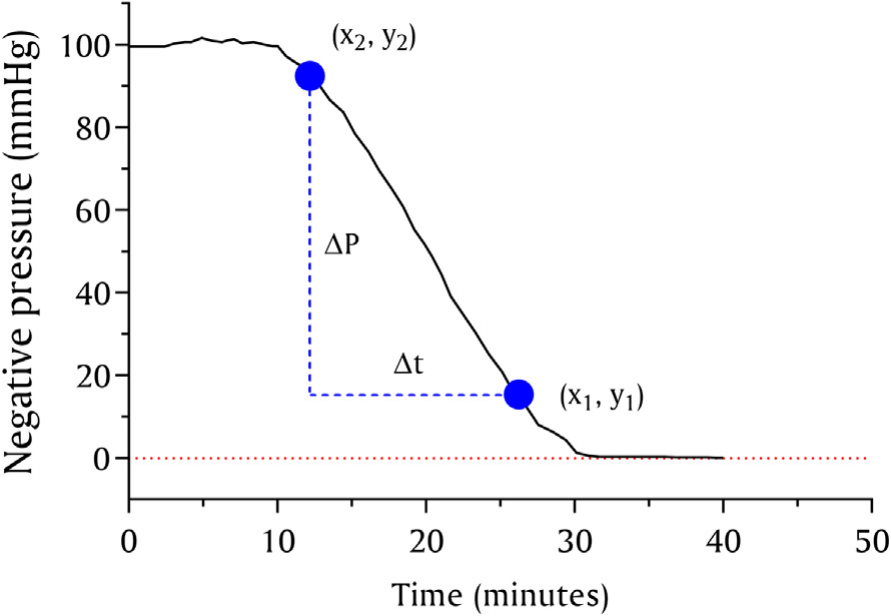
A graph showing the measurement of the *nerve stretcher’s* leakage rate.

Leakage is unavoidable in a vacuum system; for example, the leakage through the micro-vacuum pump alone is ~3 mmHg/min according to manufacturer datasheet, whereas leakage through the remaining components was unknown. We assumed the leakage rate of a *nerve stretcher* to be an aggregate of the total leakage through silicone tubing, vacuum sensor, and vacuum chamber. To test this, a vacuum of ~100 mmHg was generated in the vacuum chamber, and then the micro-vacuum pump was disconnected from its connector (P1) while the *nerve stretcher* was allowed to operate normally, with *point-A* fully closed, until the vacuum chamber reached a stable baseline as indicated by the curve reaching 0 mmHg. The test was repeated ten times (n=10), and the vacuum sensor data was retrieved, plotted, and the leakage rate was measured by finding the slope of the line using two points on the curve, as shown in Fig. 9, and found to be 5.57±0.38 mmHg/min, demonstrating that 46% of leakage is caused by components outside the micro-vacuum pump.

## 9 Conclusion

This paper presents the electronic design, manufacture, and performance testing of a *nerve stretcher*. The problems encountered with the earlier version of this device like the absence of real-time pressure monitoring in graphical mode, erroneous feedback due to vacuum sensor drift, and lack of control to operate the device remotely were subsequently addressed and reported herein. In the present design, an MCU having built-in WiFi capability and a state-of-the-art vacuum sensor are used. Additionally, a web-based user interface was employed to access *nerve stretcher’s* metrics and provide user control. The web-ui is tailored to abet some of its essential functions such as, on/off, monitoring and visualizing the pressure in real-time, setting the desired vacuum by rotating the pressure knob, and observing the movement of the animal without having physical access to the device. The positive results of the *nerve stretcher’s* performance and optimization demonstrate that it can now be employed experimentally for *in vivo* nerve stretching animal studies.

## 10 Animals ethics approval

This work has been carried out in accordance with an animal ethics protocol (NRS/01/17/AEC) approved by the Animal Ethics Committee, Griffith University, Queensland, Australia.

## Declaration of Competing Interest

The authors declare that they have no known competing financial interests or personal relationships that could have appeared to influence the work reported in this paper.

## References

[1] Sahar M.U., Barton M., Tansley G. (2019). Bridging larger gaps in peripheral nerves using neural prosthetics and physical therapeutic agents. Neural Regen. Res..

[2] Weiss P. (1968). Nerve patterns: the mechanics of nerve growth. Dyn. Dev. Exp. Inferences..

[3] Bray D. (1984). Axonal growth in response to experimentally applied mechanical tension. Dev. Biol..

[4] Smith D.H., Wolf J.A., Meaney D.F. (2001). A New Strategy to Produce Sustained Growth of Central Nervous System Axons: Continuous Mechanical Tension. Tissue Eng..

[5] Zheng J., Lamoureux P., Santiago V., Dennerll T., Buxbaum R.E., Heidemann S.R. (1991). Tensile regulation of axonal elongation and initiation. J. Neurosci..

[6] Pfister B.J., Iwata A., Meaney D.F., Smith D.H. (2004). Extreme stretch growth of integrated axons. J. Neurosci..

[7] Pfister B.J., Iwata A., Taylor A.G., Wolf J.A., Meaney D.F., Smith D.H. (2006). Development of transplantable nervous tissue constructs comprised of stretch-grown axons. J. Neurosci. Meth..

[8] Sahar M.S.U., Mettyas T., Barton M. (2019). Development of a nerve stretcher for in vivo stretching of nerve fibres. Biomed. Phys. Eng. Exp..

