# Supplementary material for: Design and fabrication of a nerve-stretching device for in vivo mechanotransduction of peripheral nerve fibers
Source: HardwareX. 2020 Feb 7;7:e00093. doi: 10.1016/j.ohx.2020.e00093 (PMC9041162; doi:10.1016/j.ohx.2020.e00093)
Supplement: Supplementary data 2 [file mmc2.zip › Schematic.pdf]

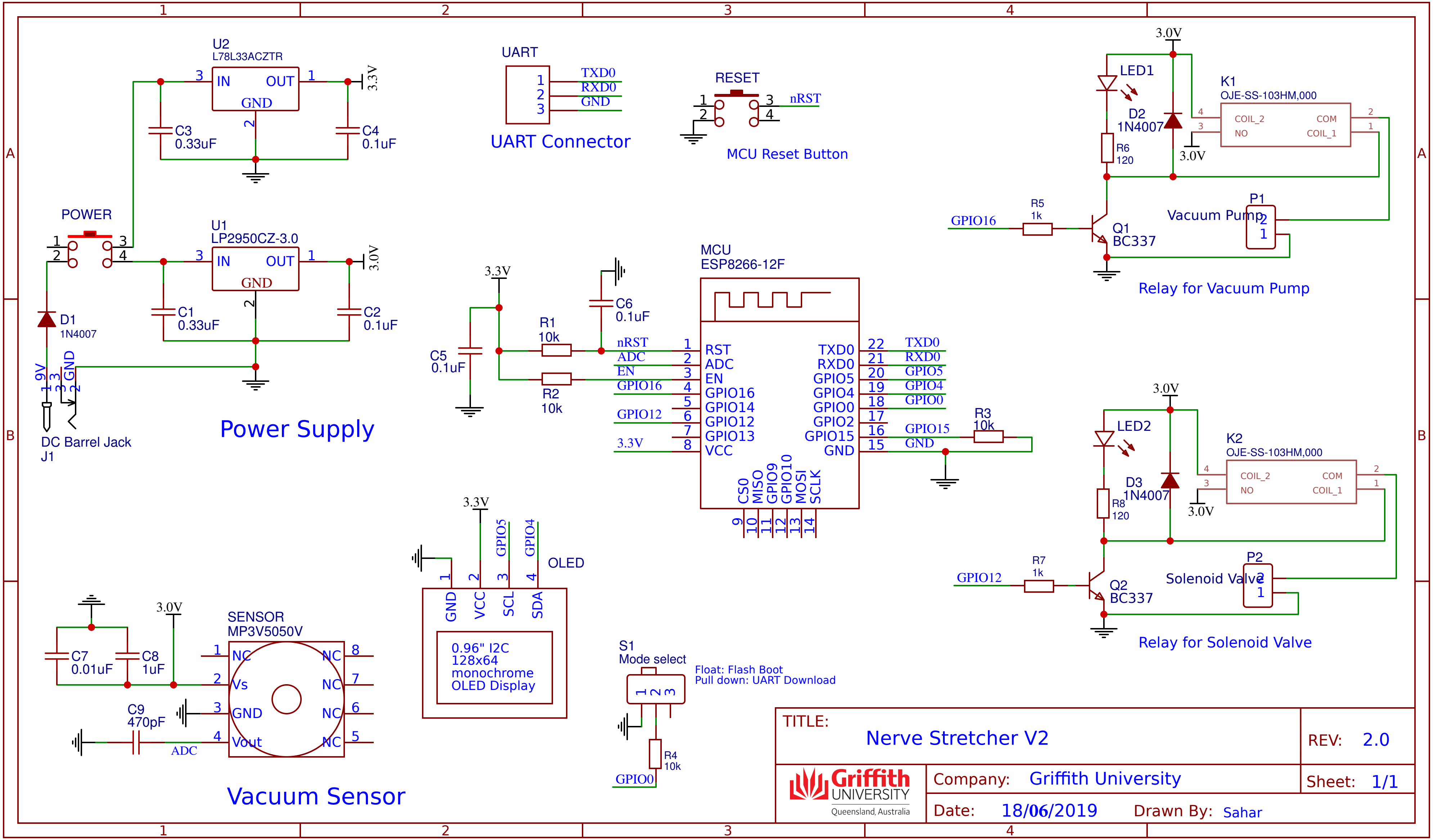

Power Supply
POWER
U2
L78L33ACZTR
IN
OUT
GND
3.3V
C3
0.33uF
C4
0.1uF
U1
LP2950CZ-3.0
3.0V
C1
0.33uF
C2
0.1uF
D1
1N4007
9V
GND
DC Barrel Jack
J1
UART
TXD0
RXD0
GND
UART Connector
RESET
nRST
MCU Reset Button
MCU
ESP8266-12F
RST
ADC
EN
GPIO16
GPIO14
GPIO12
GPIO13
VCC
TXD0
RXD0
GPIO5
GPIO4
GPIO0
GPIO2
GPIO15
GND
CS0
MISO
GPIO9
GPIO10
MOSI
SCLK
R1
10k
R2
10k
R3
10k
C5
0.1uF
C6
0.1uF
LED1
D2
1N4007
R6
120
K1
OJE-SS-103HM,000
COIL_2
COM
NO
COIL_1
P1
Vacuum Pump
R5
1k
Q1
BC337
Relay for Vacuum Pump
LED2
D3
1N4007
R8
120
K2
OJE-SS-103HM,000
P2
Solenoid Valve
R7
1k
Q2
BC337
Relay for Solenoid Valve
OLED
SDA
SCL
0.96" I2C
128x64
monochrome
OLED Display
SENSOR
MP3V5050V
NC
Vs
Vout
C7
0.01uF
C8
1uF
C9
470pF
Vacuum Sensor
S1
Mode select
Float: Flash Boot
Pull down: UART Download
R4
10k
TITLE:
Nerve Stretcher V2
REV: 2.0
Griffith UNIVERSITY
Queensland, Australia
Company: Griffith University
Sheet: 1/1
Date: 18/06/2019
Drawn By: Sahar
